# Integrin αvβ6-associated ERK2 mediates MMP-9 secretion in colon cancer cells

**DOI:** 10.1038/sj.bjc.6600480

**Published:** 2002-08-01

**Authors:** X Gu, J Niu, D J Dorahy, R Scott, M V Agrez

**Affiliations:** Newcastle Bowel Cancer Research Collaborative, Hunter Medical Research Institute, John Hunter Hospital and The University of Newcastle, NSW 2308, Australia

**Keywords:** colon cancer, integrin αvβ6, matrix metalloproteinase, MAP kinase

## Abstract

There is general consensus that matrix metalloproteinases are involved in tumour progression. We show herein that inhibition of integrin αvβ6 expression in colon cancer cells suppresses MMP-9 secretion. This integrin-mediated event is dependent upon direct binding between the β6 integrin subunit and extracellular signal-regulated kinase 2. Targetting either β6 or its interaction with extracellular signal-regulated kinase in order to inhibit matrix metalloproteinase activity may offer a useful therapeutic approach in preventing growth and spread of colon cancer.

*British Journal of Cancer* (2002) **87**, 348–351. doi:10.1038/sj.bjc.6600480 www.bjcancer.com

© 2002 Cancer Research UK

## 

Tumour progression reflects the ability of cancer cells to proliferate and invade surrounding matrix barriers and these events are regulated, at least in part, by cell adhesion receptors called integrins and matrix-degrading enzymes. Integrins consist of alpha (α) and beta (β) subunit molecules in close non-covalent association that form structural and functional bridges between the extracellular matrix and cytoskeletal proteins within a cell (Hynes, 1992). Within the αv integrin subfamily, αvβ6 is not expressed in normal epithelia; however, it becomes highly expressed during tumorigenesis and the β6 integrin subunit is thought to be widespread in cancers of the lung, breast, pancreas, ovary, oropharynx and colon as well as in the tracheal airway epithelium of heavy smokers (Sheppard *et al*, 1990; Breuss *et al*, 1995; Agrez *et al*, 1996; Thomas *et al*, 1997; Arihiro *et al*, 2000). Heterologous expression of αvβ6 in a colon cancer cell line that lacks constitutive αvβ6 expression has been shown by us to enhance tumour growth *in vitro* and *in vivo* thought to be due, in part, to αvβ6-mediated matrix metalloproteinase-9 (MMP-9) secretion (Agrez *et al*, 1994, 1999; Niu *et al*, 1998). Furthermore, we have reported that αvβ6 induces its own expression in an autocrine manner with cell crowding and proposed a self-perpetuating model of colon cancer progression regulated through αvβ6-mediated MMP-9 secretion (Niu *et al*, 2001).

The importance of the mitogen-activated protein (MAP) kinase signalling pathway in promoting cancer growth *in vivo* is now no longer in question. In a recent breakthrough in this field, a highly potent inhibitor of MAP kinase activation has been identified which is capable of inhibiting human cancer growth in immune-deficient mice (Sebolt-Leopold *et al*, 1999). We have recently demonstrated a direct physical interaction between αvβ6 and a member of the MAP kinase family, extracellular signal-regulated kinase 2 (ERK2) which defines a novel paradigm of integrin-mediated signalling in cancer (Ahmed *et al*, 2002). Moreover, we have shown that suppression of either wild-type β6 expression or expression of β6 deficient in the binding domain for ERK2 dramatically inhibits colon cancer cell growth (Ahmed *et al*, 2002). In the present study we describe the effect of either down-regulation of β6 expression or loss of the binding site on β6 for ERK2 on MMP-9 secretion.

## MATERIALS AND METHODS

### Cell lines and culture conditions

The human colon cancer cell lines WiDr and HT29 were obtained from the American Type Culture Collection (ATCC; Rockville, MD, USA) and maintained as monolayers in standard medium comprising Dulbecco's Modified Eagles Medium (DMEM; 4.5 g l^−1^ glucose) with 10% heat-inactivated foetal bovine serum (FBS) supplemented with HEPES and penicillin/streptomycin. Both cell lines were transfected with the β6 gene construct in antisense orientation and stable transfectants selected continuously in puromycin (WiDr, 1 μg ml^−1^; HT29, 2.5 μg ml^−1^) as previously described (Ahmed *et al*, 2002). Cells transfected with the expression plasmid only were established as controls (mock transfectants). Stably transfected SW480 colon cancer cells (ATCC) expressing either wild-type β6 or β6 cytoplasmic domain deletion mutant lacking the ERK2 binding site were prepared as previously described (Agrez *et al*, 1994; Cone *et al*, 1994). SW480 cells transfected with the expression plasmid only (mock transfectants) were prepared as controls (Agrez *et al*, 1994).

Tumour-conditioned medium (TCM) for MMP-9 estimation was prepared by removal of FBS-containing medium and three washes of the adherent cells with phosphate-buffered saline before addition of chemically defined serum-free medium. Serum-free medium comprised DMEM (minus phenolphthalein) supplemented with ITS (insulin, selenous acid and transferrin), HEPES and penicillin/streptomycin and was harvested 48 h later. In some experiments the MEK-1 inhibitor, PD98059 (50 μM; Calbiochem, San Diego, CA, USA) was added to the serum-free cultures. The TCM was cleared of cells and debris by centrifugation at 3290 g for 10 min, followed by protein estimation using the BCA protein assay reagent (Pierce, Rockford, IL, USA) to ensure equivalent loading onto zymogram gels.

### Zymography

MMP-9 was analysed in SDS-substrate gels by adding gelatin (0.1 mg ml^−1^ final concentration) to the 10% acrylamide separating gel. TCM collected under serum-free conditions was mixed with substrate gel sample buffer (10% SDS, 50% glycerol, 25 mM Tris-HCI (pH 6.8) and 0.1% bromophenol blue), and 70 μl loaded onto the gel without prior boiling. Following electrophoresis, gels were washed twice in 2.5% Triton X-100 for 30 min at room temperature to remove the SDS. Gels were then incubated at 37°C overnight in substrate buffer containing 50 mM Tris HCl and 5 mM CaCl_2_ (pH 8.0). Gels were stained with 0.15% Coomassie blue R250 in 50% methanol, 10% glacial acetic acid for 20 min at room temperature and de-stained in the same solution without Coomassie blue. Gelatin-degrading enzymes were identified as clear bands against the blue background of stained gel.

### MMP-9 activity assay

MMP-9 levels in TCM obtained from antisense β6 transfectants were assayed using a commercially available kit, the Biotrak MMP-9 activity assay system (Amersham, Aylesbury, UK). This assay measures total MMP-9 levels (inactive pro-enzyme activated artificially plus endogenous active enzyme forms) and MMP-9 secretion is calculated on a per-cell basis.

## RESULTS

### Suppression of β6-expression, MEK inhibition, or deletion of the β6-ERK2 binding site inhibits MMP-9 secretion

**Figure 1:**
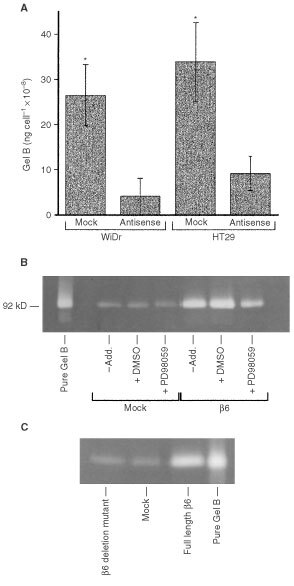
Effect of β6 suppression, MEK inhibition and deletion of the ERK2 binding site on β6 on MMP-9 secretion. (**A**) MMP-9 levels in tumour-conditioned medium (TCM) from WiDr and HT29 mock/antisense β6 transfectants. The data represent mean (±s.e.m.) levels of MMP-9 (ng cell^−1^) for three independent experiments. Asterisks denote statistically significant differences in MMP-9 secretion levels between mock and antisense β6 transfectants for each cell line (*P*<0.05, Two-sample Wilcoxin rank-sum (Mann-Whitney) test). (**B**) Gelatin zymogram showing gelatinase activity in TCM from SW480 cells transfected with either the β6 gene construct or with vector alone (mock transfectants). The cells had been exposed to either the MEK-1 inhibitor PD98059 (50 μM) or vehicle control (DMSO) and the position of purified MMP-9 is shown on the left. (**C**) Gelatin zymogram showing gelatinase activity in TCM from SW480 cells expressing either mutant β6 that lacks the ERK2 binding domain, wild-type β6 or vector alone (mock transfectants). The position of purified MMP-9 is shown on the right.

The inhibitory effect of down-regulation of β6 expression on MMP-9 secretion for the WiDr and HT29 cell lines which constitutively express αvβ6 is shown in Figure 1A

. Amounts of MMP-9 secreted per cell into serum-free tumour-conditioned medium from both lines was 2–3-fold higher for mock transfectants, compared with cells transfected with antisense β6 (Figure 1A). Transfection of SW480 cells with the β6 gene construct has been shown to markedly enhance MMP-9 secretion (Niu *et al*, 1998; Agrez *et al*, 1999). As shown in Figure 1B, SW480 β6 transfectants not only secrete markedly more MMP-9 into tumour-conditioned medium than SW480 cells transfected with vector alone (mock transfectants) but this was inhibitable by the MEK-1 inhibitor, PD98059. Repeated estimation of MMP-9 secretion by PD98059-treated cells using gel zymography and densitometric quantitation for three separate experiments revealed a reduction in MMP-9 secretion of 41±8% (mean±s.e.m., data not shown). We have recently identified the binding site on the β6 cytoplasmic domain for ERK2 (Ahmed *et al*, 2002). In the present study, heterologous expression of a β6 mutant lacking the binding site for ERK2 reduced MMP-9 secretion to levels seen for non-β6 expressing mock transfectants as shown in Figure 1C.

## DISCUSSION

Matrix degradation by MMPs is crucial for growth, invasion, metastasis and angiogenesis of tumours and increased tissue expression of MMP-9 has been observed in the progression from benign to malignant colonic epithelium (Kossakowska *et al*, 1996). Maximal expression of MMPs has also been noted at the invading margin of tumour cell islands as in colon cancer (Hewitt *et al*, 1991) and plasma MMP levels are significantly elevated in patients with this disease (Zucker *et al*, 1995). We have previously reported a direct positive correlation between levels of expression of the β6-integrin subunit in SW480 colon cancer cells and MMP-9 secretion (Agrez *et al*, 1999). Moreover, tumour cell proliferation within a three-dimensional collagen matrix in β6-expressing SW480 cells is associated with conversion of tumour cell colonies from compact to spreading colonies and exposure of the cells to a specific MMP inhibitor abolishes β6-mediated tumour cell proliferation and colony spreading within a collagen matrix (Agrez *et al*, 1999). These findings are consistent with the observation by Manasseh *et al* (1999) that transfection of a human MMP-9 vector into SW480 wild-type cells resulted in enhanced cell migration and invasion *in vitro*.

The role of MAP kinases in the regulation of MMP expression in malignant cells is now well recognised. MMP-9 production has been shown to be directly dependent on the activation of endogenous ERK signalling in hepatocyte growth factor- or epidermal growth factor-stimulated human epidermal keratinocytes (Zeigler *et al*, 1999). Induction of MMP-9 promoter activity by oncogenic Ras in squamous carcinoma cells has been shown to be abrogated by blocking the ERK 1/2 pathway (Gum *et al*, 1997). Increased transcriptional activity of the MMP-9 promoter in Ras-transformed ovarian carcinoma cells has also been shown to be mediated by MAP kinases (Gum *et al*, 1996). In the present study, we show that down-regulation of constitutive β6 expression using an antisense approach against β6, that is known to suppress MAP kinase activity (Ahmed *et al*, 2002), dramatically reduced MMP-9 levels in tumour-conditioned medium. Inhibition of MAP kinase activity by the MEK-1 inhibitor PD98059, also markedly reduced MMP-9 secretion in cells transfected with the β6 gene construct.

To specifically examine the role of β6-bound ERK2 on MMP-9 secretion we tested the ability of cells expressing a β6 deletion mutant that lacks the binding site for ERK2 to secrete MMP-9 into tumour-conditioned medium. We have previously reported that transfection of SW480 cells with either the wild-type β6 gene construct or a β6 construct lacking the binding domain for ERK2 results in equivalent levels of expression of these receptors in the respective cell lines (Ahmed *et al*, 2002). However, lack of the ERK2 binding site on β6 reduced MMP-9 secretion to levels seen for non-β6 expressing cells. This may account, at least in part, for the reduced tumour growth *in vivo* observed for cells expressing β6 that lacks the ERK2 binding site compared with cells expressing wild-type β6 (Ahmed *et al*, 2002). Importantly, in cells that either lack β6 or express β6 lacking the ERK2 binding domain, ERK2 associates with the β5 integrin subunit (Ahmed *et al*, 2002). While the significance of the β5-ERK2 binding event remains to be determined we have postulated that a hierarchy of integrin-ERK2 interactions exists within cancer cells with preferential binding of the kinase to the growth-promoting β6 subunit when it is expressed (Ahmed *et al*, 2002). It is this preferential binding event that is responsible for enhanced MMP-9 secretion in cells when they express αvβ6.

Attempts to inhibit tumour progression by blocking matrix-degrading activity have led to the development of synthetic MMP inhibitors that have shown promise in recent clinical trials involving cancers of the pancreas, stomach, lung and bowel (Wojtowicz-Praga *et al*, 1998; Primrose *et al*, 1999; Rosemurgy *et al*, 1999; Tierney *et al*, 1999). However, significant side effects from the use of MMP inhibitors have also been documented (Wojtowicz-Praga *et al*, 1998; Hutchinson *et al*, 1998; Primrose *et al*, 1999). Given that the β6-ERK2 interaction mediates MMP-9 secretion and that *de novo* expression of β6 occurs in epithelial cancer cells, inhibition of MMP activity by targetting either β6 or the ERK2 binding site may offer greater therapeutic specificity in cancer treatment than MMP inhibitors and avoid unwanted side effects.
